# Concurrent or Sequential Hormonal and Radiation Therapy in Breast Cancer: A Literature Review

**DOI:** 10.7759/cureus.364

**Published:** 2015-10-25

**Authors:** Matthew J Cecchini, Edward Yu, Kylea Potvin, David D'souza, Michael Lock

**Affiliations:** 1 Department of Pathology, Schulich School of Medicine & Dentistry, Western University, London, Ontario, CA; 2 Department of Radiation Oncology, London Regional Cancer Program, London, Ontario, CA; Schulich School of Medicine & Dentistry, Western University, London, Ontario, CA.; 3 Department of Medical Oncology, London Regional Cancer Program, London, Ontario, CA; Schulich School of Medicine & Dentistry, Western University, London, Ontario, CA; 4 Department of Radiation Oncology, London Regional Cancer Program, London, Ontario, CA; Schulich School of Medicine & Dentistry, Western University, London, Ontario, CA.

**Keywords:** breast cancer, tamoxifen, aromatase inhibitor, radiation therapy, lung fibrosis, breast fibrosis, timing of therapy

## Abstract

Background and objectives: Adjuvant hormonal therapy is frequently used in the treatment of women with estrogen receptor (ER)/progesterone receptor (PR) positive breast cancer. When radiotherapy is given, hormone therapy may be delivered in a concurrent or sequential manner. Hormonal blockade with tamoxifen or aromatase inhibitors is thought to arrest hormonally dependent cancer cells in the early G1 phase of the cell cycle. This has been theorized to reduce the efficacy of radiation, which is known to be more effective in cells that are actively dividing. Therefore, there has been a reluctance by many to treat with concurrent hormonal and radiation therapy.

Methods: We performed a search of the Medline database that led to the identification of 39 studies. Abstract and full-text review of these studies led to the identification of seven English non-review studies in peer-reviewed literature between 1995 and 2015 that addressed the question of timing of radiation and hormonal therapy. Outcome measures were captured from each of the studies.

Results: No difference in survival or local-regional recurrence was identified between concurrent versus sequential treatment. Furthermore, no difference in cosmetic outcome or adverse effects was noted for either approach. However, when comparing radiation alone or radiation and hormonal therapy, there was an increased risk of breast and lung fibrosis with combined treatment.

Conclusions: Hormone therapy, concurrent or sequential, with radiation results in comparable disease-related outcomes, including survival and recurrence. However, given the theoretical reduction in efficacy and increased rates of fibrosis with concurrent use, it is reasonable to support the use of sequential therapy.

## Introduction and background

Breast cancer is the most common cancer affecting women and accounts for 26% of newly diagnosed cancers in Canada, excluding non-melanocytic skin cancers [1]. Of these cancers, over 80% will express either the estrogen or progesterone receptor and be amenable to hormonal therapy [2]. The use of tamoxifen in the adjuvant setting is associated with a significant reduction in breast cancer recurrence and improved overall survival [3]. In similar large multicentre level I trials, aromatase inhibitors have been shown to have a disease-free survival benefit in post-menopausal women [4]. Breast-conserving surgery has been shown to have equivalent outcomes to mastectomy when combined with radiation therapy and has become the main treatment method for breast cancer patients [5]. Thereby, there are a substantial number of women who receive radiation and hormonal therapy.

Estradiol activates proliferation through transcriptional activation of c-Myc and cyclin D, which allow for downstream activation of the cyclin-dependent kinases required for progression from G1 into S phase of the cell cycle [6]. This activity of estrogen is required for the proliferation of the cancer cells; tamoxifen or aromatase inhibitors are utilized to block this pathway [6]. Treatment of cells with tamoxifen or aromatase inhibitors results in an accumulation of cells in the G1 phase of the cell cycle. Radiation sensitivity depends on the stage of the cell cycle, with cells in G2/M being the most sensitive to radiation changes [7]. Therefore, it is possible that hormonal therapy may reduce the efficacy of radiation by arresting the cells in a stage of the cell cycle that is more resistant to DNA damage.

Cell culture studies have provided conflicting results on the role of concurrent hormonal therapy and radiation. Early studies found a protective effect between hormonal therapy and radiation that corresponded with an arrest of the cells in the G1 phase of the cell cycle [8-10]. However, more recent studies have suggested a synergistic effect between tamoxifen and letrozole in enhancing apoptosis induced by radiation [11-12]. It is not clear how to reconcile the conflicting results from culture models and how these translate to the efficacy of breast cancer treatment in women.

Animal models have suggested that there may be an increased risk of lung fibrosis with concurrent treatment of hormonal therapy and radiation [13-14]. The proposed mechanism for this effect is through TGFb as tamoxifen has been shown to increase the levels of TGFb [15] and higher levels have been associated with increased rates of fibrosis, cardiac damage, and pneumonitis [16-18]. The effect on lung fibrosis appears to be limited to tamoxifen as concurrent aromatase inhibitors were not associated with an increase in lung fibrosis in a rat model [14]. Despite these theoretical contraindications, both concurrent and sequential treatment regimes are used in practice and major clinical trials.

## Review

### Methods

A systematic review was performed investigating the timing of hormonal therapy and radiation therapy in breast cancer. Only peer-reviewed studies in English involving human subjects were included. The Medline database was searched for relevant studies between 1995 and 2015. The following search strategy was employed:

breast neoplasms/radiotherapy[mh] AND breast neoplasms/surgery[mh] AND (breast neoplasms/drug therapy[mh] OR antineoplastic agents, hormonal[mh] OR tamoxifen[mh] OR aromatase inhibitors[mh]) AND ((concurrent*[tw] OR concomitant*[tw]) AND sequential*[tw]). 

Further directed searches were performed for literature related to breast and lung fibrosis and combined hormonal and radiation. References within these publications were reviewed for relevant trials. Breast Disease Site Team members were asked to provide relevant publications not included in the literature review.

### Tamoxifen and the timing of radiation

There have been three retrospective studies that have addressed the question of the timing of tamoxifen with radiation [19-21]. The data from these studies was generated from retrospective reviews of patients that received adjuvant radiation after breast-conserving surgery and either concurrent or sequential tamoxifen. As shown in Table 1, the studies contained between 278 and 500 patients with follow-up that ranged from 8.6 years to 10.4 years. In these studies, tamoxifen was given according to institutional practices, typically 20 mg daily for five years. Radiation was given with the majority of patients receiving between 45-50 Gy of radiation with an optional boost to the tumor bed to a median total dose of 64 Gy. Many of the patients also received adjuvant chemotherapy as detailed in Table 1. Given the long follow-up required to appropriately assess for breast cancer outcomes, many of these patients were treated over 30 years ago; however, this means they were treated with radiation techniques and chemotherapy regimes that are no longer the standard of practice today.

**Table 1:** Overview of studies comparing concurrent versus sequential tamoxifen and radiation in breast cancer.

Reference	Type	Pts (n)	Treatment Groups	Tamoxifen	Radiation	Chemotherapy (n)	Duration of Follow-up	Outcome
Ahn, 2005 [21]	Retrospective 1976-1999	500	Concurrent (254) vs. Sequential (241)	According to institutional practises generally for 5 years	48 Gy in 2 Gy fractions with boost to primary tumor bed median total dose 64 Gy	CMF (71) adriamycin based (42) other (16) none (371)	10.4 years	No difference in overall survival HR, 1.234;95% CI, 0.42 to 2.05 No difference in local recurrence HR, 0.932;95% CI, 0.42 to 2.05;
Harris, 2005 [19]	Retrospective 1980-1995	278	Concurrent (174) vs. Sequential (104)	20 mg OD or 10 mg BID	tangents only (182) or tangents and nodal (95) median total dose 64 Gy	methotrexate -based (67) doxorubicin-based (43) none (167)	8.6 years	No difference in overall survival HR, 1.56; 95% CI, 0.87 to 2.79 No difference in local recurrence HR, 1.22;95% CI, 0.33 to 4.49 No difference in complications or cosmesis
Pierce, 2005 [20]	Retrospective 1989-1993	309	Concurrent (202) vs. Sequential (107)	20 mg daily for 5 years	45-50 Gy to whole breast unknown dose as boost	CMF (156) CAF (153)	10.3 years	No difference in overall survival HR, 0.84; 95% CI, 0.40 to 1.78; No difference in local recurrence HR, 0.73; 95% CI, 0.26 to 2.04 No difference in Grade 3 or 4 haematological toxicities No Grade 4 pulmonary toxicity, one Grade 3 toxicity in concurrent

All of the studies quantified treatment outcomes for patients and no difference in overall survival or local recurrence was observed in any of the studies [19-21]. A pooled hazard ratio for the rate of ipsilateral recurrence was 0.91 with 95% CI 0.52 to 1.61 [22]. This analysis included 1,082 patients followed for over 10 years. Furthermore, two of the studies assessed for complications and found no difference in the rates of complications; however, Pierce, et al. noted one Grade 3 lung toxicity, and this was observed in the concurrent group with none in the sequential group [20].

### Aromatase inhibitors and the timing of radiation

Four studies have addressed the question of sequential or concurrent aromatase inhibitors with radiation and are shown in Table 2 [23-26]. Azria, et al. randomized 150 patients with low-stage breast cancer treated with breast-conserving surgery to either concurrent or sequential letrozole with whole breast radiation [23]. The primary endpoint of this study was early and late side-effects based upon physical exam and patient reported outcomes. All patients received 50 Gy in 2 Gy fraction with a boost of up to 16 Gy and 2.5 mg of letrozole daily starting either three weeks prior to radiation (concurrent arm) or after radiation (sequential arm) [23]. Of the 150 patients in the study, 28 patients received adjuvant FEC chemotherapy [23]. No difference was found in the rate of early or late side-effects, including subcutaneous fibrosis or lung fibrosis [23]. No difference was observed in quality-of-life measures for either group [23]. The study was relatively small with only 75 patients in each of the arms with a limited follow-up of 2.2 years. Clinical outcomes, such as local recurrence, were not addressed due to the limited follow-up; however, additional follow-up is planned to address the question of survival and local recurrence [23].

**Table 2:** Overview of studies comparing concurrent versus sequential aromatase inhibitors and radiation in breast cancer

Reference	Type	Pts (n)	Treatment Groups	Tamoxifen	Radiation	Chemotherapy (n)	Duration of Follow-up	Outcome
Valakh, 2009 [26]	Retrospective 1998-2008	183	Concurrent (57) vs. Sequential (126)	anastrozole or tamoxifen	45-54 Gy with a 1-1.6 Gy boost over an average of 49.5 days	anthracycline or taxane (51) none (132)	2.3 (Con) 2.6 (Seq)	No difference in dermatitis or fibrosis Local recurrence 4% in sequential and 1.8% in concurrent
Ishitobi, 2009 [24]	Retrospective 2001-2008	278	Concurrent (113) Vs. Sequential (151)	anastrozole 1mg (270) letrozole 2.5 mg (8) for 5 years	50 Gy in 2 Gy fractions with a boost of up to 63.2 Gy for positive margins	CMF (1) taxane based (7) anthracycline based (31) anthracycline and taxane (6) none (233)	2.9	No recurrences or deaths in either group No difference in Grade 3 or 5 toxicities
Azira, 2010 [23]	Randomized 2005-2007	150	Concurrent (75) Vs. Sequential (75)	2.5 mg daily for 5 years starting 3 weeks before (Seq) or after (Con) Radiation	50 Gy in 2 Gy fractions with a boost of up to 16 Gy median dose 60 Gy	FEC (28) none (122)	2.2	No difference in acute or late side effects No difference in subcutaneous fibrosis No difference in lung fibrosis No difference in quality of life
Ishitobi, 2014 [25]	Retrospective 2001-2009	315	Concurrent (158) Vs. Sequential (157)	anastrozole 1mg (301) letrozole 2.5 mg (14) for 5 years	50 Gy in 2 Gy fractions with a boost of up to 63.2 Gy for positive margins	yes (57) none (258)	5.6	No difference in disease-free survival Non-significant increase in deaths without recurrence in concurrent group 3 patients vs. 0 patients p=0.08 No difference in Grade 3 or 5 toxicities

A number of retrospective reviews have also addressed the sequence of aromatase inhibitors with radiation. Ishitobi has published two reports on this topic in 2009 and 2011 with an overlap of patients between the two studies [24-25]. The studies involved patients treated between 2001 and 2009 with a follow-up of 5.1 years in the most recent study [25]. The majority of patients received 1 mg of anastrozole for five years and 50 Gy of radiation in 2 Gy fractions with a boost of up to 63.2 Gy for positive margins. The majority of patients in the study did not receive chemotherapy with incomplete reporting on the type of chemotherapy in the most recent study [24-25]. Valakh also reported a retrospective study of 183 patients treated with sequential or concurrent hormonal therapy that consisted of either anastrozole or tamoxifen [26]. In all the studies, no difference in Grade 3-5 toxicities were noted [24-25]. In the more recent Ishitobi, et al. study with a longer follow-up of 5.1 years, outcome data was presented with no difference in overall survival or local recurrence [24-25]. However, a non-significant increase in deaths without recurrence was noted in the concurrent group with three patients versus zero in the sequential group (p=0.08) [25]. No significant difference in local recurrence was observed in the Valakh, et al. study [26].

### Breast and lung fibrosis

The question of breast fibrosis and combined hormonal therapy and radiation has been addressed in a number of retrospective studies (Table 3) [27-33]. All studies compared women treated with radiation alone to women with combined radiation and tamoxifen. Johansen, et al. performed a retrospective analysis of patients involved in a randomized trial comparing breast-conserving surgery with mastectomy [34-35]. High-risk postmenopausal patients that received adjuvant tamoxifen were compared to the low-risk group that only received radiation [31]. The median follow-up for patients was 6.6 years with objective scores of breast and skin changes scored by the oncologist and cosmetic outcomes independently scored by the patient and the oncologist [31]. A significant difference in Grade 2 or greater fibrosis was noted in the radiation and tamoxifen group (19% vs. 48%); however, this did not translate into a statistically significant difference in the cosmetic outcome as reported by the patient or the oncologist [31].

**Table 3:** Overview of studies outlining breast fibrosis with adjuvant radiation and hormonal therapy

Reference	Type	Pts (n)	Treatment Groups	Hormonal Agent	Radiation	Chemotherapy (n)	Duration of Follow-up (Years)	Outcome
Wazer, 1992 [28]	Retrospective (1982-1988)	234	examined all patients treated for prognostic markers for cosmesis	Tamoxifen 10 mg BID (22)	Cobalt-60 or 6MV linear accelerator treated to 50-50.4 Gy With boost to for positive margins with external beam or Iridium implants	CMF (56) CMF or CAF (22) None (156)	4.2	Non-significant trend towards worse cosmetic outcome in patients treated with tamoxifen and radiation
Taylor, 1995 [29]	Retrospective (2001-2008)	456	examined all patients treated for prognostic markers for cosmesis	Tamoxifen (76)	Cobalt-60 or 4-6MV linear accelerator treated to 45-50.4 Gy with boost to margins in some cases	CMF or CAF (95) None (348)	2.9	No difference in cosmetic outcome for patients treated with tamoxifen
Fowble, 1996 [32]	Retrospective (1982-1991)	491	tamoxifen (154) No tamoxifen (337)	Tamoxifen 10 mg BID for a minimum of 2 years	46-50Gy over 4.5-5 weeks with 6MV linear accelerator. Boost to primary site with electrons, external beam or Iridium implants	None (491)	5.3	No difference in cosmetic outcome Increase in breast edema in tamoxifen group (49% vs. 31%)
Markiewicz, 1996 [30]	Retrospective (1977-1991)	1053	No adjuvant (419) Chemotherapy (105) tamoxifen (105) Chemotherapy and hormonal therapy (56)	Tamoxifen	45-50 Gy with the primary tumor bed boosted with electrons or Iridium	CMF or CAF	6.7	No difference in cosmetic outcomes
Wazer, 1997 [27]	Retrospective (1982-1994)	498	tamoxifen (130) No tamoxifen 368	Tamoxifen 20 mg daily	Cobalt-60 or 6MV linear accelerator treated to 50-50.4 Gy With boost to for positive margins	CAF or CMF (124) None (374)	4.7 years (tamoxifen group) 5 years for no tamoxifen	No significant difference in cosmesis
Azria, 2004 [33]	Retrospective	147	RT Alone (57) vs. RT + tamoxifen (90)	Tamoxifen 20 mg daily	50 Gy with 6MeV boost of 10-16 Gy to surgical bed	None	2.4	Increased incidence of Grade 2 or 3 subcutaneous fibrosis with tamoxifen
Johansen, 2007 [31]	Retrospective	96	RT alone (69) vs. RT and tamoxifen (27)	Tamoxifen	48-50 Gy with a boost of 10 Gy to the tumor bed	None	6.6	Higher rate of fibrosis of Grade 2 or greater 19% vs. 48% P=0.004 in tamoxifen group

Azria, et al. performed a retrospective analysis of patients involved in a prospective trial measuring the predictive value of CD4 and CD8 T-lymphocyte apoptosis for predicting late side-effects of radiation therapy. Of the 147 patients treated with radiation alone or tamoxifen, a significant difference in the incidence of Grade 2-3 subcutaneous fibrosis was observed in the combined tamoxifen and radiation group [33]. Fowble, et al. did not evaluate skin complications, but found an increase in breast edema in patients treated with tamoxifen and radiation. Fowble found no difference in cosmetic outcomes [32]. After a multivariate analysis, Wazer, et al. did not find a difference in cosmesis for patients treated with adjuvant tamoxifen and radiation [27-28]. This is further supported by two other similar studies that did not find any association with cosmetic outcomes [29-30]. Taken together, an increased rate of low-grade fibrosis has been observed with concurrent radiation and hormone therapy, but this has not translated into differences in cosmetic outcomes.

A number of prospective studies ranging from 1996 to 2011 have measured the rates of lung fibrosis post-treatment with tamoxifen and radiation using serial imaging by chest x-ray or CT scan (Table 4) [36-38]. In all three studies, an increased risk of pulmonary fibrosis was detected in patients with combined radiation and tamoxifen treatment compared to radiation alone. However, the majority of the fibrosis was Grade 1 or Grade 2 with limited symptoms and only detected on imaging. In the Bentzen, et al. study, patients were accrued between 1978 and 1982 and were post-mastectomy patients treated with anterior 8-MV photon field covering the axillary, infraclavicular, and supraclavicular areas; these patients were followed by serial x-rays [38]. The techniques utilized in this study are no longer part of the standard of care so caution must be used in applying the results of this study to current populations. Koc, et al. studied post-mastectomy patients treated between 1996 and 2001 with Cobalt-60 radiation to the chest wall and lymphatics and followed patients with serial CT scans to quantify pulmonary fibrosis [37]. The authors followed 111 women and found an increase in pulmonary fibrosis in 26/74 of patients treated with tamoxifen compared with 5/37 treated with radiation alone [37]. There was a significant difference in patients with Grade 2 or Grade 3 fibrosis in the tamoxifen and radiation arm; however, only two of the patients required treatment with steroids [37]. Varga, et al. included patients treated with both breast-conserving surgery and mastectomy treated with modern tangential techniques and found no difference in symptomatic or non-symptomatic pneumonitis. As in the Koc study, Varga found an increase in Grade 1 fibrosis as detected on serial follow-up CT scans [36].

**Table 4:** Overview of studies outlining lung fibrosis with adjuvant radiation and hormonal therapy

Reference	Type	Pts (n)	Treatment Groups	Tamoxifen	Radiation	Chemotherapy (n)	Duration of Follow-up (Years)	Outcome
Bentzen, 1996 [38]	Prospective Randomized	84	Radiation therapy plus tamoxifen (38) VS radiation alone (46) Followed with serial Chest X-ray	tamoxifen 10 mg TID for 48 weeks	Anterior 8-MV photon field 26.6-51.4 Gy	No chemotherapy	Min 5	Increased risk of pulmonary fibrosis (RR= 2.0; 95% CI 1.2-3.5; P = .01)
Koc, 2002 [37]	Prospective (1996-2001)	111	Post-mastectomy radiation cobalt receiving concurrent tamoxifen VS radiation alone​ Followed with serial CT Scans	tamoxifen 20 mg daily for 5 years	50Gy in 2Gy fractions	CAF (73) CMF (27) CE (4) Taxol adriamycin cyclophosphamide (2) none (5)	3-3.45	Increased Pulmonary fibrosis in 26/74 of tamoxifen + RT VS 5/37 with RT alone. (P= 0:01)
Varga, 2011 [36]	Prospective (2001-2004 and 2006-2008)	328	tamoxifen (77) AI (82) or no adjuvant (90) Chemotherapy (79)​ Followed with serial CT scans	tamoxifen 20mg daily anastrozole 1mg daily​ letrozole 2.5 mg daily	20 Gy in 2 Gy fractions	taxane based (79) 249 (90)	1	Increased rate of Grade 1 pulmonary fibrosis for tamoxifen (OR=2.0 (1.02–3.9, p=0.041) No difference in pneumonitis for tamoxifen​ No Difference in fibrosis of pneumonitis for AI

In the studies comparing sequential to concurrent treatment regimes, no change in lung toxicity was noted. In the Harris, et al. study, no difference was noted in pneumonitis between the two groups as based on identification by the radiation oncologist; fibrosis detected on follow-up imaging was not reported [19]. The rates of fibrosis were not described in Pierce, et al., but one Grade 3 pulmonary toxicity was noted in the concurrent group compared to the sequential group [20].

There is a lack of data on the potential synergistic effects between hormonal therapy and adverse effects to the heart. The TGFb cytokine has been shown to be involved in fibrosis of the heart [39]. As such, it is reasonable to postulate that a similar relation exists with concurrent treatment and potential fibrosis to the heart. In early animal models, no additional toxicity appears to be imparted by concurrent tamoxifen therapy with radiation [40]. However, this important question has yet be fully explored in clinical models and warrants further analysis to ensure that no increased toxicity is imparted to the heart by combining radiation and hormonal therapy.

### Discussion

The question of the timing of hormonal therapy is an important one as small changes in the relative risk of clinical outcomes could impact on a large absolute number of patients, given the high incidence of this hormone-sensitive sub-group. In the absence of clear guidelines regarding the timing of these two therapies, there is a significant degree of heterogeneity in the treatment of patients. From a theoretical standpoint, there is a proposed contraindication of hormonal therapy concurrent with radiation due to the anti-proliferative effects of hormonal treatments and a decreased efficacy of radiation on arrested cells. Furthermore, animal models suggest that tamoxifen can increase the levels of TGFb [15], leading to increased levels of fibrosis in involved radiation fields [16-17]. This is contrasted with some literature that suggests that there may be a synergistic effect between aromatase inhibitors and radiation [11-12]. Taken together, there are opposing rationales for concurrent versus sequential treatment regimes. From a practical point of view, a consistent recommendation is desired by patients and caregivers.  A consistent recommendation would avoid contradicting recommendations to a patient by treating physicians, patients’ losing confidence in their treating physicians, and the possibility of patients missing the initiation of treatment.

Medical oncologists have also wrestled with this issue with several contradictory results. Studies such as Bedognetti, et al. found no differences in survival and toxicity events when 431 patients were randomized in a multicenter trial to sequential or concurrent hormonal treatment and chemotherapy [41]. SWOG 8814 randomized 1,558 postmenopausal node-positive patients to concurrent and sequential treatment, plus a variation in chemotherapy using a three-arm design. Sequential treatment was found to have better disease-free survival (HR 0.76 95%CI 0.64-0.91;p=0.002) and a non-significant improvement in overall survival [42]. So despite varied data, medical oncologists have long concluded that sequential treatment for chemotherapy is the standard of care. This has been based on the lack of any evident or perceived benefit for concurrent administration and possible harm. This has also been applied to aromatase inhibitors, which are also given sequentially despite limited data [43]. This practical approach has been used as a guide to address the same question in this radiation oncology literature review.

Another issue is the chance that hormonal therapy will be missed and patients with ER/PR-positive disease will not receive this beneficial treatment due to the uncertainty that exists with the timing of radiation and hormonal therapy. In the interplay between the medical and radiation oncologists, there is potential for one or the other to assume that the other will be starting hormonal therapy, especially if the timing of treatment is variable. For example, the medical oncologist could hold tamoxifen treatment in a patient that is scheduled to start radiation to avoid perceived risks of concurrent treatment with the assumption that the radiation oncology team will start treatment upon completion. If this rationale is not documented, it is conceivable that the radiation oncologist would assume that the medical oncologist has already discussed tamoxifen with the patient and decided to not use tamoxifen therapy or was already on adjuvant hormonal management. Therefore, there is a need for clear communication and consensus in this area of uncertainty for both practical and theoretical reasons.

Sequential treatment may have a theoretical impact on compliance. There is evidence that there is a poor compliance with hormonal therapy, and this is associated with increased mortality in women with breast cancer [46-47]. A large retrospective study looking at compliance with hormone therapy found that the addition of radiation or chemotherapy were both associated with an increased rate of non-compliance with hormone therapy on univariate analysis [48]. Given that the adverse effects of tamoxifen or AIs are worst in the first three months, the additional of side-effects imparted by radiation may be intolerable to patients and promote decreased compliance. Therefore, the sequential approach may enhance patient compliance and warrants further investigation.

There is no evidence that concurrent or sequential tamoxifen or aromatase inhibitors alter treatment outcomes for patients treated with radiation [19-21, 25-26]. However, these studies may not have the power to detect a difference, given that the majority of studies are retrospective in nature and, especially in the case of aromatase inhibitors, may have insufficient follow-up. Further, the retrospective studies were conducted, in some cases, on patients treated over 30 years ago. These patients were treated with radiation techniques and chemotherapy regimes that are not currently used for our current patients. As such, the results may not be directly applicable to patients treated with modern techniques. However, this should have improved the ability to detect a difference as failure rates would have been higher and the impact of small changes in treatments, such as sequencing of therapy, would be more noticeable. Ideally, large randomized trials with the power to detect potential differences in the timing of radiation and endocrine therapy would be beneficial. Currently, there is an effort to address this question in the CONSET trial (NCT00896155), a large randomized trial ongoing in India opened in 2009 [44].

No significant differences in clinically adverse outcomes were observed in the studies that looked at the timing of hormonal therapy with radiation [19-20, 24-26]. While differences were detected in studies that addressed the question of breast fibrosis [27-33] or lung fibrosis [36-38] with or without radiation, these studies were not designed to detect clinically important outcomes. Further, the majority of fibrosis was low-grade and did not lead to a change in cosmetic outcomes, cardiac events, or symptomatic lung disease. Therefore, while there may be a signal for increases in fibrosis with tamoxifen and radiation, the clinical significance of this is uncertain. This further underscores the lack of randomized data with significant follow-up to measure adverse events associated with modern techniques and treatment modalities. As the sample size required to detect this difference is likely to be very large, we may have to rely on literature reviews and consensus opinions. Support for further long-term follow-up of existing studies and reliance on larger database cohorts to monitor for late events that may be associated with treatment are an important means to answer this question. In the situation of potentially small differences in outcome, population-based studies can be considered more effective to answer questions of safety and efficacy [45].

## Conclusions

There is no clear evidence to suggest that either concurrent or sequential hormonal and radiation therapy results in a change in clinically important outcomes or adverse events. However, there is literature that suggests that concurrent radiation and hormonal therapy may enhance lung, soft-tissue, and cardiac fibrosis through increased levels of TGFb. It is conceivable that sequential hormonal therapy and radiotherapy may avoid these toxicities. Taken together, it is reasonable for patients to complete hormonal therapy and radiation in a sequential fashion to limit the risk of fibrosis without sacrificing oncologic outcomes. However, due to the limited nature of the trials, this conclusion must be considered with caution.
